# A longitudinal cohort study of malaria exposure and changing serostatus in a malaria endemic area of rural Tanzania

**DOI:** 10.1186/s12936-017-1945-2

**Published:** 2017-08-02

**Authors:** Ryan A. Simmons, Leonard Mboera, Marie Lynn Miranda, Alison Morris, Gillian Stresman, Elizabeth L. Turner, Randall Kramer, Chris Drakeley, Wendy P. O’Meara

**Affiliations:** 1 Department of Biostatistics and Bioinformatics, Duke University Medical Center, 2721, Durham, NC 27701 USA; 2 Duke Global Health Institute, Duke University, 310 Trent Drive, Durham, NC 27701 USA; 3 National Institute for Medical Research, 3 Barack Obama Drive, P.O. Box 9653, 11101 Dar es Salaam, United Republic of Tanzania; 4 Department of Statistics, Rice University, Houston, TX USA; 5 Department of Immunology and Infection, London School of Hygiene and Tropical Medicine, Keppel Street, London, WC1E 7HT UK

**Keywords:** Serology, AMA-1, MSP-1, Malaria

## Abstract

**Background:**

Measurements of anti-malarial antibodies are increasingly used as a proxy of transmission intensity. Most serological surveys are based on the use of cross-sectional data that, when age-stratified, approximates historical patterns of transmission within a population. Comparatively few studies leverage longitudinal data to explicitly relate individual infection events with subsequent antibody responses.

**Methods:**

The occurrence of seroconversion and seroreversion events for two *Plasmodium falciparum* asexual stage antigens (MSP-1 and AMA-1) was examined using three annual measurements of 691 individuals from a cohort of individuals in a malaria-endemic area of rural east-central Tanzania. Mixed-effect logistic regression models were employed to determine factors associated with changes in serostatus over time.

**Results:**

While the expected population-level relationship between seroprevalence and disease incidence was observed, on an individual level the relationship between individual infections and the antibody response was complex. MSP-1 antibody responses were more dynamic in response to the occurrence and resolution of infection events than AMA-1, while the latter was more correlated with consecutive infections. The MSP-1 antibody response to an observed infection seemed to decay faster over time than the corresponding AMA-1 response. Surprisingly, there was no evidence of an age effect on the occurrence of a conversion or reversion event.

**Conclusions:**

While the population-level results concur with previously published sero-epidemiological surveys, the individual-level results highlight the more complex relationship between detected infections and antibody dynamics than can be analysed using cross-sectional data. The longitudinal analysis of serological data may provide a powerful tool for teasing apart the complex relationship between infection events and the corresponding immune response, thereby improving the ability to rapidly assess the success or failure of malaria control programmes.

## Background

Malaria imposes a severe public health burden in tropical and sub-tropical regions, particularly sub-Saharan Africa [1]. Quantifying the impact of control strategies on exposure and infection is essential to improving and scaling-up effective strategies [2]. This is often accomplished by monitoring changes in incidence, prevalence and/or entomological estimates of malaria transmission intensity [3]. One promising alternative approach is the use of serological measures of malaria exposure, which involves detecting antibodies produced by the body in response to an infection in a plasma or serum sample.

Anti-malarial antibody responses not only contribute to mediating protection from the disease, but also represent a marker of individual, and population, exposure history. Since antibodies persist longer in the human body than the parasite itself (longer, too, than the lifespan of the vector), serological markers may represent more robust and sensitive measurements of transmission intensity than entomological or parasitological markers [4–6]. The utility of sero-epidemiological surveillance has been demonstrated by numerous studies in Tanzania [7, 8], Vanuatu [9], Equatorial Guinea [10], Brazil [11], and Uganda [12]. The most widely studied markers include antigens to the *Plasmodium falciparum* apical membrane antigen 1 (AMA-1) and merozoite surface protein 1 (MSP-1_19_) recombinant proteins, both of which have been proposed as malaria vaccine candidates [13].

Serology has been used as a tool to measure transmission intensity and monitor changes as far back as the 1950s. Nearly all examples of this approach employ cross-sectional serological surveys coupled with serocatalytic models to estimate the average seroconversion and seroreversion rates in the study population [7, 14]. Serocatalytic models use age as a proxy for historical time, treating each individual’s observed serostatus as “a random realization of a seroconversion-seroreversion stochastic law” [14]. These models often make a number of important assumptions, namely: that the risk of seroconversion is directly proportional to individual exposure; that individual serostatus is a relatively stable state over time; and that seroreversion rates are stable over time, meaning that a fixed proportion of people will seroconvert as well as serorevert every year. These are assumptions that can only be tested in a longitudinal setting.

There have been comparatively few longitudinal assessments of malaria serologic data [15–21], each with important limitations. Several studies were conducted in very low transmission settings with fewer than a dozen seroconversion events [15, 19], several studies followed participants for less than a year [19, 20], and only one study included older children and adults [18]. Bejon et al. [22] followed a cohort of individuals in Kenya for several years; however, the analysis was restricted to children under 15 years of age, and conducted separately within each survey year rather than longitudinally. None of these studies investigated individual predictors of seroconversion or seroreversion events, or examined the dynamics of the antibody response following a detectable infection.

There is a relative lack of longitudinal data aimed at elucidating medium or long-term trends in antibody response to malaria in a large study population. Such insights are required to improve the use of serological outcomes in monitoring malaria transmission within large-scale control programmes. Current approaches have been validated to infer longitudinal trends in malaria transmission based on cross-sectional data but nuances in transmission dynamics may be missed [4, 7, 8]. The richer longitudinal data of the Mvomero study in Tanzania [23] was leveraged to examine the temporal dynamics of serostatus and the roles of age and malaria parasitaemia in shaping these dynamics. The present study specifically explores some of the assumptions that underlie cross-sectional, population-based serological analysis, including the relationship between serostatus and age, the nature of seropositive state, and rates of seroreversion over time.

## Methods

### Data sources

A longitudinal, cluster-randomized study was conducted in 24 randomly selected villages in Mvomero district in rural east-central Tanzania from 2011 to 2013. In a two-stage design, villages were enrolled as clusters and households randomly sampled from within each village; six study villages were assigned to each of four groups (control; a disease management strategy involving early detection and treatment by community health workers using rapid diagnostic technology; vector control through community-supported larviciding; and early detection and treatment plus larviciding). A cohort of 5385 people from 962 households (approximately 40 households per village) were enrolled and sampled in three consecutive years during the long rainy season, for a total of 16,155 possible measurements. The first survey was conducted in March and April 2011, with follow-up conducted in the same months of 2012 and 2013. A detailed description of the study design can be found in Kramer et al. [23].

This analysis was restricted to the sub-set of individuals for whom parasitological and serological outcomes, for either AMA-1 or MSP-1, were available at all three time points; namely, those with complete-case data. There were 681 individuals with complete-case data for AMA-1, and 686 individuals with complete-case data for MSP-1. There were five individuals with complete information for AMA-1 data who were missing at least one MSP-1 measurement, and there were ten individuals with complete MSP-1 data who were missing at least one AMA-1 measurement. As a consequence, there were a total of 691 individuals in the current evaluation. The age ranges, and other relevant characteristics, of these individuals are shown in Table 1.

**Table 1:** Characteristics of Mvomero study population with complete case serology data (N = 691)

	Survey 1 (2011)	Survey 2 (2012)	Survey 3 (2013)
Age in years (N%)			
<2	145 (21.0%)	101 (14.6%)	30 (4.3%)
2–5	149 (21.6%)	166 (24.0%)	173 (25.0%)
5–10	129 (18.7%)	134 (19.4%)	173 (25.0%)
10–15	31 (4.5%)	53 (7.7%)	74 (10.7%)
15–20	18 (2.6%)	14 (2.0%)	18 (2.6%)
20–30	88 (12.7%)	82 (11.9%)	69 (10.0%)
30–40	81 (11.7%)	79 (11.4%)	81 (11.7%)
40+	50 (7.2%)	62 (9.0%)	73 (10.6%)
Gender			
Male	272 (39.4%)		
Female	419 (60.6%)		
Malaria infections			
N (%)	34 (5.0%)	47 (6.8%)	95 (14.2%)
Seropositive (AMA-1)			
N (%)	313 (45.4%)	249 (36.4%)	302 (43.9%)
Seropositive (MSP-1)			
N (%)	227 (33.0%)	192 (27.9%)	278 (40.2%)
Number of seroconversion events (AMA-1)	n/a		
N (% of seronegative in previous survey)		19 (5.0%)	73 (19.7%)
Number of seroconversion events (MSP-1)			
N (% of seronegative in previous survey)		42 (9.1%)	110 (22.0%)
Number of seroreversion events (AMA-1)			
N (% of seropositive in previous survey)		81 (25.9%)	21 (8.4%)
Number of seroreversion events (MSP-1)			
N (% of seropositive in previous survey)		77 (33.9%)	26 (13.5%)

### Parasitological measures

At each annual data collection round, participants provided a finger-prick blood sample from which a dried blood spot was stored and a malaria smear was prepared. Blood spots were stored with desiccant at −20 °C prior to and after shipping to the London School of Hygiene and Tropical Medicine, with laboratory analyses conducted on all samples at the conclusion of the study. Thick and thin blood smears were stained with Giemsa solution and examined with a binocular microscope with an oil immersion lens to quantify the parasitaemia. Parasitaemia was measured by counting the number of asexual parasites against the number of leukocytes in the blood film, based on a count of 8000 leukocytes per microlitre. The number of asexual parasites was counted against 200 leukocytes using a hand tally counter. A slide was considered negative if no malaria parasite was observed in at least 200 oil-immersion fields.

### Serological measures

Antibodies to malaria antigens were detected from serum samples of study participants using indirect enzyme-linked immunosorbent assays (ELISA). The technique is described fully in Stewart et al. [8]. Briefly, plasma is eluted from dried blood spots. The concentration (or titre) of the antibody (measured in arbitrary units, AU, per microlitre) is inferred by the optical density values recorded from an ELISA reader. Raw optical density values are converted into an estimated titer using a standard curve generated by titration of a positive control sample on each assay plate. This analysis focused on antibodies to the *P. falciparum* apical membrane antigen 1 (AMA-1; 3D7 strain) and merozoite surface protein 1 (MSP-1_19_; Wellcome genotype) recombinant proteins, which were produced as described [24, 25]. All samples were processed and analysed together at the conclusion of the study to reduce systematic variation that can arise between batches of reagents.

### Data analysis

Two different classes of models were fitted: one using the continuous antibody titre as the outcome, and another using observed (binary) seroconversion or seroreversion events as the outcome. Separate models of each class were fitted for AMA-1 and MSP-1 (for a total of six models). Observed seroconversion events were defined as when an individual who was seronegative in one survey year became seropositive in the following year; observed seroreversion events were defined as when an individual who was seropositive in one survey year become seronegative in the following year. As such, with data from three consecutive years, it is only possible to identify these events in the final 2 years (2012 and 2013). Serostatus (i.e., seropositive or seronegative) was determined by designating a titre threshold, above which a sample was deemed to be seropositive, and below which was deemed to be seronegative. For each antigen, this threshold was determined by fitting a two-component normal mixture model to the estimated titer distribution; the threshold was calculated as the mean of the ‘seronegative’ (i.e. lower titer) component plus three standard deviations [5].

The continuous antibody titre models were fitted as normal mixed-effects models [26] with natural log-transformed antibody titres as the outcome and individual-level random intercepts accounting for the correlation within individuals over time. For the seroconversion/seroreversion models, the outcome was a binary indicator of whether or not a seroconversion or seroreversion event was observed in 2012 or 2013. The model was fitted as a logistic regression with random intercepts at the individual level. Each individual could only contribute a single positive outcome (i.e., could only seroconvert or serorevert once) to each model because there were only two follow-up time periods, making the comparison between individuals who experienced an event vs those who never experienced that event (e.g., an individual who seroconverted in 2012 did not contribute to the model for 2013, since they had already seroconverted).

In both classes of models, lasso variable selection was used to find the most parsimonious model [27]. The following set of possible predictors was entered: age, presence or absence of an observed malaria infection (either concurrent with the outcome event or in the preceding year), and serostatus for the alternate antibody (i.e., MSP-1 as a predictor when AMA-1 is the outcome, and vice versa), both concurrent with the outcome event and in the previous year. In addition, an indicator variable for year (2012 vs 2013) was included to account for differences in the number of events between survey years. The two-way interactions for all predictors listed above were included in the model selection procedure. The relationship between cross-sectional seroprevalence and age is known to follow a logistic growth curve, often modelled using a reversible catalytic conversion (RCC) model [7, 28]. To account for the non-linear relationship between age and seropositivity, the exposure-driven relationship with age was modelled as a linear spline with a knot at age 20. Exploratory analyses determined this to be a reasonable approximation, with the spline expressing a similar value for the concordance (*c*) statistic [29] compared to the corresponding RCC model for both antibodies (for AMA-1: spline *c* = 0.85, RCC *c* = 0.84; for MSP-1: spline *c* = 0.72, RCC *c* = 0.72). In practice, after implementing the lasso approach for each outcome and for both antibodies, all two-way interactions were dropped from each of the six final models. All analyses were performed using SAS/STAT software, version 9.4 of the SAS System for Windows. Copyright © 2013 SAS Institute Inc., Cary, NC, USA.

## Results

### Descriptive statistics

The characteristics of the 691 individuals in the analysis sample are shown by survey year in Table 1. Most (423 individuals, 61.3% of the sample) were below the age of 10 years at baseline; due to aging, this shifted to 376 (54.4%) by the third survey. Relatively few people in this cohort were between the ages of 10 and 20 years, with 49 individuals in 2011 (7.1%), rising to 92 (13.3%) by 2013. One-hundred and thirty-nine individuals (20.1%) had at least one malaria infection during the study; 34 individuals were missing a malaria status in at least one survey year. Of the 176 total observed malaria infections, 34 (19.3%) were in 2011, 47 (26.7%) were in 2012, and 95 (54.0%) were in 2013. There were 92 observed seroconversion events for AMA-1 and 152 for MSP-1 (corresponding to 13.5 and 22.1% of individuals, respectively). For both antibodies, the majority (79.3 and 72.3%, respectively) of seroconversion events were observed in 2013. Additionally, there were 102 observed seroreversion events for AMA-1 and 103 for MSP-1, with the majority (79.4 and 74.8%, respectively) being observed in 2012.

Individuals were classified by observed malaria infection ‘trajectory’-in other words, their malaria infection status across time in each survey year; the characteristics of individuals within each trajectory group are shown in Table 2. Thirty-four individuals with missing parasitaemia data were excluded. Of the 139 individuals with at least one detected malaria infection, 27 (19.4%) had multiple detected infections during the follow-up period. These 27 were, on average, younger than those with one or zero observed infections. The proportion of seropositive individuals was greater amongst participants with at least one observed infection (0.72 for AMA-1 and 0.68 for MSP-1; N = 139) than among those never infected (0.49 for AMA-1 and 0.45 for MSP-1; N = 518).

**Table 2:** Characteristics of study population by malaria trajectory (N = 657)

	000 (N = 518)	001 (N = 69)	010 (N = 24)	100 (N = 19)	011 (N = 13)	101 (N = 6)	110 (N = 4)	111 (N = 4)
Age at baseline								
Mean (SD)	14.77 (16.06)	13.94 (13.95)	13.61 (13.49)	21.79 (22.73)	8.00 (8.40)	12.50 (10.39)	6.75 (3.77)	8.00 (4.24)
Gender								
Male	194 (37.5%)	32 (46.4%)	11 (45.8%)	8 (42.1%)	8 (61.5%)	2 (33.3%)	1 (25.0%)	4 (100.0%)
Female	324 (62.5%)	37 (53.6%)	13 (54.2%)	11 (57.9%)	5 (38.5%)	4 (66.7%)	3 (75.0%)	0 (0.0%)
Seropositive at any time point (AMA-1)								
N (%)	251 (48.5%)	47 (68.1%)	15 (62.5%)	14 (73.7%)	10 (76.9%)	6 (100.0%)	4 (100.0%)	4 (100.0%)
Seropositive at any time point (MSP-1)								
N (%)	231 (44.6%)	49 (71.0%)	20 (83.3%)	14 (73.7%)	6 (46.2%)	4 (66.7%)	2 (50.0%)	0 (0.0%)

*0* no detectible infection, *1* detected infection (e.g., 001 denotes no detectible infection in 2011 and 2012, then an infection detected in 2013; etc.)

Table 3 shows the characteristics of the analysis population by serostatus trajectory (i.e., individual patterns of serostatus over time; if 0 = seronegative and 1 = seropositive, then an individual who was never observed to be seropositive during the study has a trajectory of 000. An individual who was only seropositive in 2011 would have a trajectory of 100, etc.). For both antibodies, individuals who were seropositive at multiple time points (e.g., 110) were, on average, older than individuals who were never seropositive. Malaria infection prevalence was greater among individuals who were seropositive in at least one time point (AMA-1: 101/367 or 27.5%; MSP-1: 99/344 or 28.8%) compared to individuals who were seronegative at all time points (AMA-1: 41/314 or 13.1%; MSP-1: 45/342 or 13.2%).

**Table 3:** Characteristics of study population by serostatus trajectory

	AMA-1 (N = 681)							
	000 (N = 314)	001 (N = 40)	010 (N = 12)	100 (N = 47)	011 (N = 7)	101 (N = 33)	110 (N = 9)	111 (N = 219)
Age at baseline								
Mean (SD)	5.21 (7.20)	11.52 (17.13)	14.18 (16.01)	17.48 (15.71)	16.79 (24.50)	24.35 (17.34)	30.33 (18.41)	26.59 (14.93)
Gender								
Male	147 (46.8%)	21 (52.5%)	2 (16.7%)	17 (36.2%)	3 (42.9%)	14 (42.4%)	4 (44.4%)	59 (26.9%)
Female	167 (53.2%)	19 (47.5%)	10 (83.3%)	30 (63.8%)	4 (57.1%)	19 (57.6%)	5 (55.6%)	160 (73.1%)
Infected at any time point								
N (%)	41 (13.1%)	10 (25.0%)	3 (25.0%)	6 (12.8%)	5 (71.4%)	8 (24.2%)	0 (0.0%)	69 (31.5%)

	MSP-1 (N = 686)							
	000 (N = 342)	001 (N = 75)	010 (N = 17)	100 (N = 43)	011 (N = 25)	101 (N = 34)	110 (N = 9)	111 (N = 141)
Age at baseline								
Mean (SD)	10.18 (12.70)	10.49 (15.41)	6.81 (9.89)	13.81 (14.60)	7.64 (9.89)	20.40 (17.16)	34.94 (19.73)	28.50 (15.14)
Gender								
Male	152 (44.4%)	36 (48.0%)	8 (47.1%)	17 (39.5%)	11 (44.0%)	11 (32.4%)	3 (33.3%)	34 (24.1%)
Female	190 (55.6%)	39 (52.0%)	9 (52.9%)	26 (60.5%)	14 (56.0%)	23 (67.6%)	6 (66.7%)	107 (75.9%)
Infected at any time point								
N (%)	45 (13.2%)	28 (37.3%)	8 (47.1%)	5 (11.6%)	8 (32.0%)	5 (14.7%)	2 (22.2%)	43 (30.5%)

*0* seronegative, *1* seropositive (e.g. 001 denotes seronegative in 2011 and 2012, then seropositive in 2013; etc.)

**Fig. 1:**
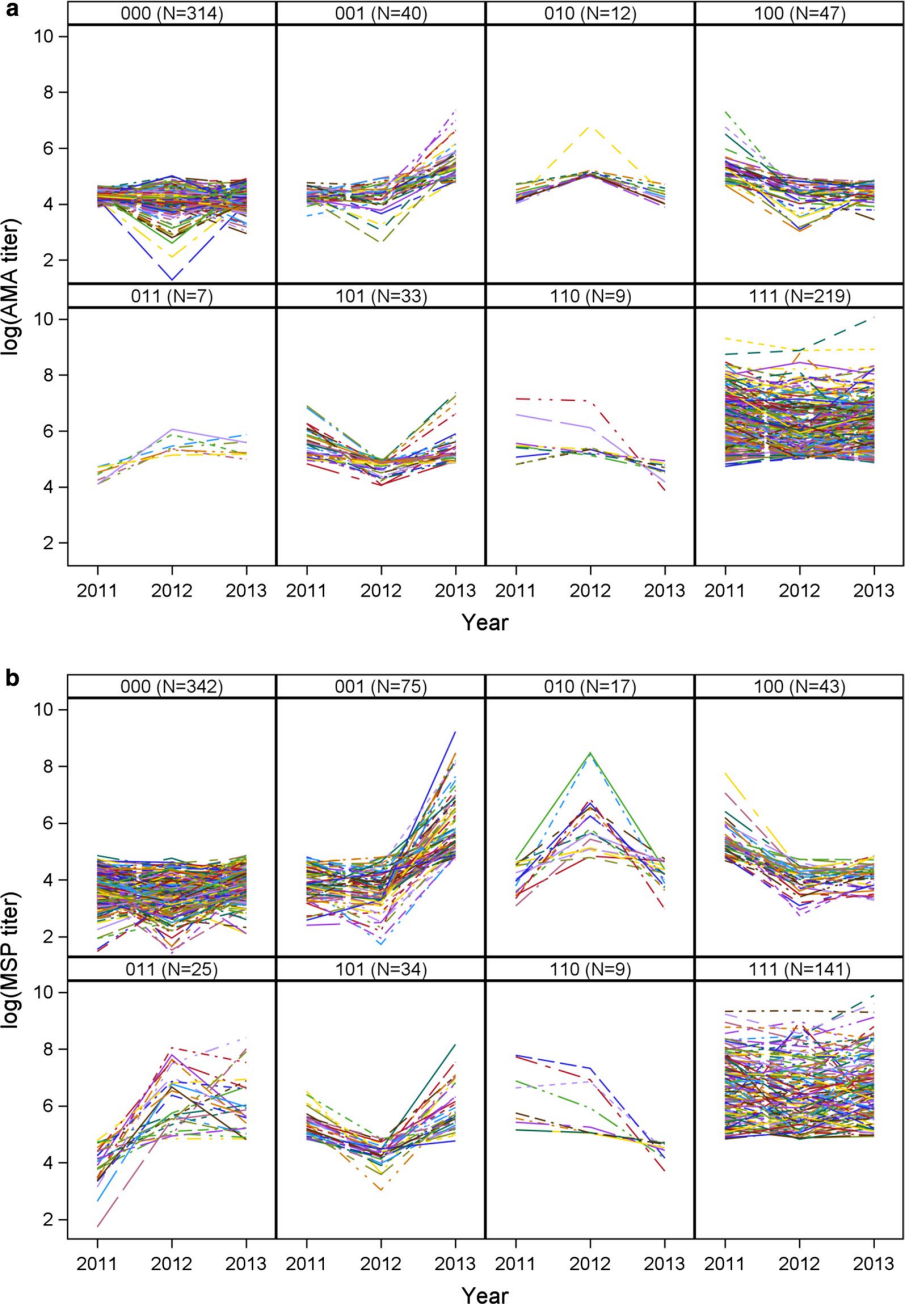
Individual log titre trajectories across survey years. For AMA-1 (**a**) and MSP-1 (**b**) by serostatus classification (*0* seronegative,* 1* seropositive; e.g. *001* denotes seronegative in 2011 and 2012, then seropositive in 2013, etc.)

Individual log-titre values over time within each serostatus trajectory for both antibodies are shown in Fig. 1. In general, the titre levels across time reflect the derived serostatus trajectory; however, it is clear that using binary serostatus in a longitudinal framework does not capture the heterogeneity in the actual titre. Although seropositive individuals had a greater degree of variability in their absolute titre values, seronegative individuals also had a high level of variation especially if these individuals are interpreted as malaria-naïve or unexposed.

**Fig. 2:**
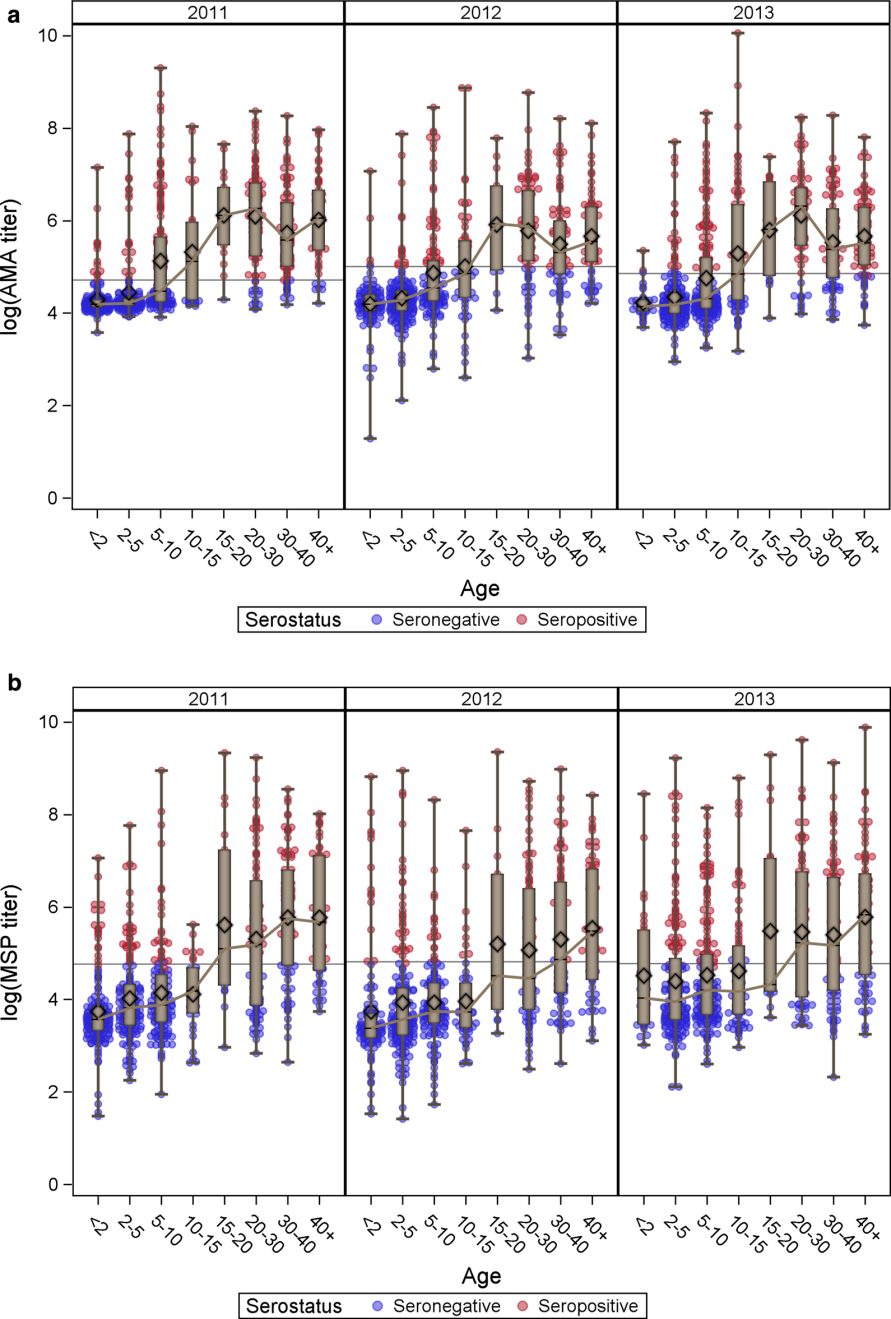
Age-stratified seroprevalence curves in each survey years. For AMA-1 (**a**) and MSP-1 (**b**). The* points* denote an individual’s log-titre value for the respective antibody in the given survey year, colored* red* for titres classified as seropositive and* blue* for titres classified as seronegative. The* solid lines* connect the median log-titre within each age group

The distribution of log-titre by serostatus as a function of age within each survey year is shown in Fig. 2. The relationship between age and median titre (denoted on the plots by the solid line) roughly matches the logarithmic growth of seroprevalence over age observed in cross-sectional surveys [7]. Of particular interest is the difference between the age trends for AMA-1 and MSP-1 (panel A vs panel B). The slope is sharper for AMA-1 than for MSP-1, with an apparent decline within the oldest strata of age (though this may in part be driven by a smaller sample size within that strata). This is consistent with previously reported differences in seroprevalence for these antigens [7, 20].

### Individual seroconversion event models

Individuals who were seronegative in one year and seropositive the following year (i.e., in 2011 and 2012, or in 2012 and 2013) were defined as having seroconverted. Those who seroconverted were compared to those who remained seronegative in a mixed-effects logistic regression. Table 4 shows the odds ratios for seroconverting for AMA-1 and MSP-1. Individuals had 3.6 [95% confidence interval (CI) 2.1–6.2] times greater odds of seroconverting for AMA-1 in 2013 than in 2012, and 2.8 (95% CI 1.9–4.3) times greater odds for MSP-1. Age did not significantly impact individual odds of seroconverting for AMA-1, but did for MSP-1: individuals 20 years of age and younger were slightly less likely to seroconvert (OR 0.93, 95% CI 0.89–0.97) while individuals older than 20 were slightly more likely to seroconvert (OR 1.08, 95% CI 1.02–1.16). Individuals who seroconverted to the alternate antigen were significantly more likely to seroconvert to the outcome antigen, compared to individuals who remained seronegative across both years to the alternate antigen: MSP-1 seroconversion (in a given year) was associated with 2.79-fold (95% CI 1.64–4.76) higher odds of AMA-1 seroconversion in that same year, while AMA-1 seroconversion was associated with 3.93-fold (95% CI 2.26–6.81) higher odds of MSP-1 seroconversion. Sero*reversion* to the alternate antigen, on the other hand, was associated with significantly reduced odds of seroconversion to the outcome antigen (compared to individuals who remained seronegative across both years to the alternate antigen): the odds of seroconverting for AMA-1 was multiplied by 0.25 (95% CI 0.13–0.50) when a seroreversion was observed for MSP-1 compared to those who did not experience a seroreversion. The odds ratio of seroconverting for MSP-1 when a seroreversion for AMA-1 was observed in the same year was 0.50 (95% CI 0.28–0.90). Being seropositive in both years for MSP-1 did not impact the odds of AMA-1 seroconversion (OR 0.70, 95% CI 0.36–1.34) compared to being seronegative in both years for MSP-1. However, for those who were being positive for AMA-1 in both years, the odds of MSP-1 seroconversion was multiplied by 1.97 (95% CI 1.10–3.51), compared to being negative in both years.

**Table 4:** Odds ratios (and 95% CIs) from mixed-effects logistic regression models for each outcome (seroconversions and seroreversions) on each antibody (AMA-1 and MSP-1)

Outcome		
Parameter	Seroconversion	Seroreversion
	OR (95% CI)	OR (95% CI)
AMA-1		
Year (2013 vs 2012)	*3.56 (2.06, 6.15)*	*0.31 (0.19, 0.53)*
Age (≤20)	0.99 (0.95, 1.04)	*1.06 (1.02, 1.10)*
Age (>20)	1.04 (0.97, 1.11)	0.95 (0.90, 1.01)
Malaria in current year	1.34 (0.70, 2.54)	0.73 (0.28, 1.91)
Malaria in previous year	2.07 (0.94, 4.56)	0.51 (0.15, 1.71)
MSP-1 seropositive in current year	*2.79 (1.64, 4.76)*	*0.31 (0.16, 0.58)*
MSP-1 seropositive in previous year	*0.25 (0.13, 0.50)*	*2.01 (1.15, 3.51)*
MSP-1		
Year (2013 vs 2012)	*2.84 (1.89, 4.25)*	*0.43 (0.26, 0.79)*
Age (≤20)	*0.93 (0.89, 0.97)*	1.00 (0.96, 1.05)
Age (>20)	*1.08 (1.02, 1.16)*	1.01 (0.95, 1.08)
Malaria in current year	*2.70 (1.65, 4.42)*	0.98 (0.45, 2.09)
Malaria in previous year	*0.37 (0.15, 0.95)*	1.95 (0.89, 4.25)
AMA-1 seropositive in current year	*3.93 (2.26, 6.81)*	*0.39 (0.21, 0.71)*
AMA-1 seropositive in previous year	*0.50 (0.28, 0.90)*	*2.58 (1.39, 4.81)*

Italicized cells indicate statistical significance

In the AMA-1 model, neither concurrent (OR 1.34, 95% CI 0.70–2.54) or preceding year (OR 2.07, 95% CI 0.94–4.56) malaria infection status (at the time of sampling) was a significant predictor of seroconversion, compared to individuals without an infection in either year. However, individuals who had a malaria infection in both years had 2.77 (95% CI 1.11–6.89) times higher odds of seroconverting compared to individuals without an infection in either year. By contrast, in the MSP-1 model, those who did not have an observed malaria infection in the preceding year but did have one in the concurrent year were significantly *more* likely to MSP-1 seroconvert than individuals with no observed malaria infections in either year (OR 2.70, 95% CI 1.65–4.42). Individuals who did not have a malaria infection in the concurrent year but did in the preceding year were significantly *less* likely to MSP-1 seroconvert (OR 0.37, 95% CI 0.15–0.95). Having a malaria infection in *both* years was not associated with any difference in the odds of MSP-1 seroconversion (OR 1.00; 95% CI 0.38–2.68), compared to individuals with no observed infections.

### Individual seroreversion event models

Using a similar principle as for seroconversion, a seroreversion event was defined as when an individual who was seropositive in a given year (2011 or 2012) was seronegative in the following year (2012 or 2013). Those who seroreverted were compared to those who remained seropositive in a mixed-effects logistic regression. Table 4 shows the odds ratios for the analogous seroreversion models for AMA-1 and MSP-1. For both antigens, individuals were less likely to serorevert in 2013 than in 2012 (for AMA-1: OR = 0.31, 95% CI 0.19–0.53; for MSP-1: OR 0.43, 95% CI 0.26–0.79). For AMA-1, individuals 20 years or younger in age were more likely to serorevert (OR 1.06; 95% CI 1.02–1.10). Older individuals were no more or less likely to serorevert for AMA-1, and neither age effect was significant in the MSP-1 model. Individuals who seroreverted to the alternate antigen in the same time frame were significantly more likely to serorevert to the outcome antigen, compared to individuals who remained seropositive in both years: seroreversions for MSP-1 were associated with increased odds of seroreverting for AMA-1 (OR 3.21; 95% CI 1.72–6.02) while seroreversions for AMA-1 were associated with 2.58-fold (95% CI 1.41–4.74) greater odds of seroreverting for MSP-1. Meanwhile, seroconverting to the alternate antigen was associated with reduced odds of seroreverting to the outcome antigen, compared to individuals who remained seropositive in both years: individuals who seroconverted for MSP-1 were 0.50 (95% CI 0.29–0.87) as likely to serorevert for AMA-1, while individuals who seroconverted for AMA-1 were 0.40 (95% CI 0.21–0.72) as likely to serorevert for MSP-1. In both cases, the odds of seroreversion to the outcome antigen was not impacted by remaining seronegative to the alternate antigen in both years (for AMA-1, OR 1.60, 95% CI 0.89–2.86; for MSP-1, OR 1.00, 95% CI 0.53–1.90).

Having a malaria infection in the current, previous or both years was not significantly associated with AMA-1 seroreversion compared to individuals without any observed infections. For MSP-1, having an observed malaria infection in the previous year but not the current year was associated with higher odds of seroreverting (OR 1.95; 95% CI 0.89–4.25) compared to individuals without an infection in either year, but the effect was not statistically significant. Neither concurrent malaria infections (OR 0.98; 95% CI 0.45–2.09) nor having a malaria infection in both years (OR 1.90; 95% CI 0.69–5.23) were significantly related to the odds of MSP-1 seroreversion, again compared with individuals with no observed infections.

### Continuous titre models

The continuous titre models were fitted as mixed-effects models with the natural log-transformed titre values for each antibody as the outcome. Table 5 shows the parameter estimates for the continuous antibody titre models for AMA-1 and MSP-1. Average log-titres were higher in 2013 than 2012 for both antibodies (AMA-1: 0.11, 95% CI 0.04–0.18; MSP-1: 0.45, 95% CI 0.35–0.56). Antibody titre in a given year is most strongly correlated to the titre in the previous year (AMA-1: 0.75, 95% CI 0.72–0.79); MSP-1: 0.68, 95% CI 0.64–0.72). Titre was significantly higher in participants with a concurrent malaria infection (AMA-1: 0.27, 95% CI 0.16–0.38; MSP-1: 0.29, 95% CI 0.12–0.47). Antibody titres in the *concurrent* year were correlated: a one unit increase in MSP-1 titre was associated with a 0.21 (95% CI 0.18–0.25) increase in AMA-1 titre, and a one-unit increase in AMA-1 titre was associated with a 0.53 (95% CI 0.45–0.61) unit increase in MSP-1 titre. There was an inverse relationship between outcome antibody titre and titre for the alternate antibody in the *previous* year. Log AMA-1 titre *decreased* by 0.11 (95% CI 0.07–0.14) for each one-log increase in MSP-1 titre in the previous year. A similar relationship is observed for MSP-1 titre, which decreased by 0.34 (95% CI 0.25–0.42) for each one log increase in AMA-1 titre in the previous year. The major difference between the two antibodies is the direction of the effect for observed malaria infections in the preceding year. For AMA-1, an observed malaria infection in the preceding year, but not the concurrent year, was significantly associated with higher log-titre (0.15; 95% CI 0.01–0.29). The opposite effect was observed for MSP-1 titre; malaria infection in the preceding year was associated with lower log-titre in the concurrent year (−0.34; 95% CI −0.42 to 0.25).

**Table 5:** Parameter estimates (and 95% CIs) from normal mixed-effects models for each antibody (AMA-1 and MSP-1)

Parameter	AMA-1	MSP-1
	Estimate (95% CI)	Estimate (95% CI)
Year (2013 vs 2012)	*0.11 (0.04, 0.18)*	*0.45 (0.35, 0.56)*
Malaria in current year	*0.27 (0.16, 0.38)*	*0.29 (0.12, 0.47)*
Malaria in previous year	*0.15 (0.01, 0.29)*	*−0.35 (−0.57, −0.13)*
AMA-1 log-titre in current year	n/a	*0.53 (0.45, 0.61)*
AMA-1 log-titre in previous year	*0.75 (0.72, 0.79)*	*−0.34 (−0.42, −0.25)*
MSP-1 log-titre in current year	*0.21 (0.18, 0.25)*	n/a
MSP-1 log-titre in previous year	*−0.11 (−0.14, −0.07)*	*0.68 (0.64, 0.72)*

Italicized cells indicate statistical significance

## Discussion

This study describes antibody responses to malaria antigens in samples collected over a 3-year period from a cohort in a malaria-endemic population. The profiles of these responses over time were more dynamic than anticipated: seroreversion events were just as common as seroconversion events (in fact, for AMA-1, they were more common). Further, the number of events changed dramatically between subsequent surveys, with a 300% increase in the total number of seroconversion events between 2011 and 12 and 2012 and 13 and a corresponding 70% reduction in the total number of seroreversion events over the same time period, which parallels changes in infection prevalence estimates between the surveys. In contrast, studies using cross-sectional data often assume static rates over time, especially for seroreversion [4, 7, 14, 15]. Although there are extensions of these methods designed to detect changes in the seroconversion rate at specific points in time, for model-fitting purposes these often treat the seroreversion rate as fixed [8, 30].

Despite the well-studied relationship between age and population-level seroprevalence that forms the basis of most cross-sectional models, such as the reversible catalytic conversion and superinfection models [7, 10, 30], the present analyses of individual-level changes across years found age to have very little role in predicting individual titre levels or the incidence of individual seroconversion or seroreversion events. This concords with the findings of Yman et al. [31] and Wipasa et al. [32], who both observed a lack of a distinct age effect on individual antibody titres. This implies that change in serostatus may be solely governed by age-independent exposures, and it is the accumulation of these exposures over time that results in age-dependent seroprevalence patterns, rather than the mechanisms of change themselves being a function of age. While there were few age-related effects on change in serostatus in this study, on average the individuals with multiple observed detectable infections were younger than individuals with one or fewer infections. It is possible that the greater number of infections in the youngest individuals resulted in higher antibody responses, whereas adults were either infected less frequently or had sub-microscopic infections that served to maintain antibody response levels.

As expected, on a population level, the occurrence of seroconversion and seroreversion events tracked with changes in the number of observed infection events, with higher incidence of malaria associated with a higher number of seroconversions for both antibodies [4, 10, 12, 20, 33]. The increase in seroconversion events and decrease in seroreversion events between 2011 and 2012 and 2012 and 2013 corresponded with a twofold increase in the number of observed malaria infections. However, the individual-level models demonstrated more complex dynamics at work, with different patterns and magnitudes of responsiveness for AMA-1 and MSP-1 to the introduction, resolution and sustenance of detectible infections. Seroconversion for MSP-1 was related to changes in infection status (with subsequent infections associated with greater odds and resolved infections associated with lower odds). However, seroreversion was not sensitive to such changes. Meanwhile, changes in AMA-1 serostatus were not especially sensitive to changes in infection status, but consecutive (or repeated) infections in subsequent survey years were associated with significantly elevated odds of seroconversion. Overall, MSP-1 appeared to be more sensitive to ‘short-term’ (i.e., 1 year) changes in infection status while AMA-1 was more sensitive to ‘long-term’ (i.e., 2 year) changes. However, interpretation is limited by the lack of complete infection histories for the individuals in this cohort.

The present results differ from some of the common assumptions made by cross-sectional analyses; further analysis of longitudinal serologic data may reveal more complexities that cross-sectional methods are ill suited to fully capture. For example, between 2011 and 2012, the number of observed seroreversion events for both antibodies were markedly higher than the number of seroconversion events, despite a slight *increase* in the observed number of malaria infections. This suggests the possibility that there was a change in transmission intensity not directly observable through looking at prevalence alone. The apparent relationship between seroreversion and exposure implies that changes in seroreversion rates (and not just a reduction in seroconversion rates as in many cross-sectional studies) may be a useful monitoring tool for understanding changes in exposure, particularly in the context of a highly effective prevention intervention. Further, a cross-sectional approach using standard serocatalytic models is ill suited to estimating seroreversion rates [15, 34], and would have underestimated the change in seroconversion and seroreversion rates observed between survey years.

The results from the continuous titre models support the inferences from the seroevent-based models. The increased odds of a seroconversion event, and decreased odds of a seroreversion event, between the 2012 and 2013 survey years coincides with both an increase in the average log-titre levels for both antibodies from 2012 to 2013 and a concomitant increase in the number of observed infections. It was also observed that if an individual’s log-titre increases for one antibody, it will, on average, increase for the other during the same time frame. This reflects the same phenomenon whereby if an individual seroconverts for one antibody they are more likely to seroconvert for the other. Similarly, the negative coefficient for alternate antibody in the previous year suggests that if an individual seroreverts for one antibody they are less likely to seroconvert for the other. For both AMA-1 and MSP-1, changes in serostatus were correlated, but maintaining serostatus for one antigen did not affect the odds of seroconversion or seroreversion for the other.

Concurrent malaria infections were associated with increased average log-titre levels for both antibodies. However, *previous* malaria infections were associated with significantly increased log-titre for AMA-1, but significantly decreased log-titre for MSP-1. Consecutive infection statuses were associated with significantly increased log-titre for AMA-1, but did not impact average log-titre levels for MSP-1. This would seem to imply that, while antibody titre is elevated for both during an infection, the response is preserved over a longer time frame for AMA-1 than for MSP-1, which may begin to clear more rapidly following the resolution of the infection. This would account for the differential effects of previous year infection status and consecutive infection statuses on outcomes between the AMA-1 models and the MSP-1 models. Serological and parasite based analysis may be operating on different temporal scales, something that is reflected in both the time required to mount a response and the persistence of antibodies following infection. More work remains to be done to refine the statistical methods necessary to most efficiently leverage continuous serologic data. However, this study demonstrates that the serostatus-based and titre-based models can produce complementary results in a longitudinal framework.

The stimulation of these antibodies as a response to known infection events is not well understood, and is further complicated by boosting due to repeated infections [35]. While some studies have demonstrated rapid acquisition and clearance patterns in response to clinical malaria infections [36–38], others have found responses to be more persistent over time [5, 7, 32]. The presence or absence of symptoms or parasite densities may influence the response profile of the antibodies, which may be related to acquired immunity as a function of lifetime exposure patterns [16, 20]. Although some studies have compared the results of separate models of antibody responses, the examination of the relationship between the response of different antibodies within the same model, as done in the present study, has been an underexplored area of research [17, 21].

One strength of the present study is that the continuous titre models have the outcome antibody titre in the previous year as a predictor of that antibody titre in the current year. The magnitude of that coefficient gives an estimate of the degree to which an antibody response is maintained on a year-to-year basis, controlling for both infection status at point of sampling and titre for the alternate antibody. This would seem to further support the notion that the MSP-1 response decays at a faster rate than AMA-1, since the degree to which elevated titre in the previous year is associated with current titre is reduced. Although this would contrast with the findings of Akpogheneta et al. [17], who did not identify any differences between the short-term response dynamics of AMA-1 and MSP-1, this interpretation supports the findings of White et al. [21], who calculated shorter half-lives for long-lasting antibody-secreting cells to MSP-1 compared to AMA-1, and Wipasa et al. [32].

There are several important limitations to this study. The sub-set of individuals included in the analysis (i.e., individuals with serological data at all three surveys) represents a biased sample from the overall Mvomero study population: e.g., there were more children under five and adult women used in this analysis compared to the overall sample. However, that these characteristics were not associated with any of our outcomes mitigates concerns about the influence of selection bias on the interpretation of the results [39]. Further, the number of seropositive (for both antibodies) and infection events per survey in our subset were comparable with those in the overall sample.

The classification of individuals as seropositive and seronegative makes a number of assumptions regarding the distribution of antibody titre in the population and how it relates to discrete biological states [40]. Although some of the ‘observed’ events are likely to be spurious (i.e., incurred by the assumption of a fixed titre cut-off separating distinct positive and negative sub-populations), there is no a priori reason to assume that this impacts seroreversion estimates any more or less so than it does seroconversion estimates (for example, misclassification may be expected to result in a global over- or underestimation of the rate of change of serostatus within the population, but not to preferentially misclassify individuals as having seroreverted compared to seroconverted or vice versa). Further, this is a limitation shared by other analytic frameworks for estimating population serodynamics and is not specific to this study, while the general agreement between the sero-event-based and continuous titre models assuages concerns over the validity of the dichotomization.

Finally, the present study relies on serological and parasitological measurements taken a year apart during the March–April rainy season. It is possible that this does not give a granular enough picture of individual infection or titre trajectories over time to make positive determinations about the way these trajectories are shaped by infection events. Undoubtedly, many infection events went unobserved between the sampling time-points; in addition, submicroscopic infections, which may potentially make up a large proportion of all malaria infections [41], would not have been detected in this study. However, this is no different from cross-sectional surveys that use age-stratified seroprevalence curves as proxies for cumulative exposure over time [7]. Further, more work needs to be done to understand the relevance of submicroscopic infections on malaria transmission, surveillance, and control. Despite this, the concordance of this study’s results with those reported in other studies gives confidence that these limitations do not invalidate the conclusions drawn.

## Conclusions

The potential of serologic data in malaria surveillance in a variety of transmission settings has long been acknowledged [5]. Several authors have used retrospective analyses of repeated cross-sectional surveys to track population-level changes in malaria transmission using serologic data [42–44]. Such cross-sectional analyses of malaria serology data have been proposed as a tool for surveillance, with shifts in the age-stratified seroprevalence curves used to identify the success or failure of malaria control programmes [4, 8, 43]. This approach has been shown to perform well in estimating medium and long-term trends [7, 14]. However, these cross-sectional methods use age as a substitute for time, and any judgements of a control programme’s success can only be made after cohorts of programme-exposed individuals have made their way through the population age structure. Thus, it may take years for the effect of a programme on seroprevalence to become detectible if relying solely on long-lived antigens of exposure such as AMA-1 and MSP-1. These methods are useful for providing an overview of average transmission levels in the population or for evaluating consistent and sustained changes in exposure over several years; however, they are limited in their ability to detect short term changes. They are further limited by an inability to adjust for individual-level differences in patterns of exposure over time.

Longitudinal methods, on the other hand, can model time directly; the indicator variable for survey year in the models used in these analyses may act as a direct measurement of year-to-year fluctuations in exposure across the study population. Longitudinal surveillance of events is likely to be more sensitive than cross-sectional methods, particularly to short-term changes in malaria exposure. The present study demonstrates that longitudinal serostatus and titre changes track with other metrics of transmission (e.g., prevalence), while also offering additional insight not possible through prevalence or cross-sectional methods alone (e.g., the occurrence of seroreversion events between survey years). The simultaneous modelling of multiple antigens with different response dynamics offers a novel method for potentially shedding light on the timing of changes in exposure. Further, this approach highlights the potential of the use of the continuous variable of antibody titres in repeated sampling for determining the efficacy of a programme.

## Abbreviations

AMA: apical membrane antigen
ELISA: enzyme-linked immunosorbent assays
MSP: merozoite surface protein
RCC: reversible catalytic conversion

## References

[1] WHO (2015). World malaria report 2015.

[2] Hay SI, Okiro EA, Gething PW, Patil AP, Tatem AJ, Guerra CA (2010). Estimating the global clinical burden of *Plasmodium falciparum* malaria in 2007. PLoS Med.

[3] WHO (2014). From malaria control to malaria elimination: a manual for elimination scenario planning.

[4] Corran P, Coleman P, Riley E, Drakeley C (2007). Serology: a robust indicator of malaria transmission intensity?. Trends Parasitol..

[5] Drakeley C, Cook J (2009). Potential contribution of sero-epidemiological analysis for monitoring malaria control and elimination: historical and current perspectives. Adv Parasitol.

[6] Tusting LS, Bousema T, Smith DL, Drakeley C (2015). Measuring changes in *Plasmodium falciparum* transmission: precision, accuracy and costs of metrics. Adv Parasitol.

[7] Drakeley CJ, Corran PH, Coleman PG, Tongren JE, McDonald SLR, Carneiro I (2005). Estimating medium- and long-term trends in malaria transmission by using serological markers of malaria exposure. Proc Natl Acad Sci USA.

[8] Stewart L, Gosling R, Griffin J, Gesase S, Campo J, Hashim R (2009). Rapid assessment of malaria transmission using age-specific sero-conversion rates. PLoS ONE.

[9] Cook J, Reid H, Iavro J, Kuwahata M, Taleo G, Clements A (2010). Using serological measures to monitor changes in malaria transmission in Vanuatu. Malar J..

[10] Cook J, Kleinschmidt I, Schwabe C, Nseng G, Bousema T, Corran PH (2011). Serological markers suggest heterogeneity of effectiveness of malaria control interventions on Bioko Island, Equatorial Guinea. PLoS ONE.

[11] Cunha MG, Silva ES, Sepúlveda N, Costa SPT, Saboia TC, Guerreiro JF (2014). Serologically defined variations in malaria endemicity in Pará state, Brazil. PLoS ONE.

[12] Lynch C, Cook J, Nanyunja S, Bruce J, Bhasin A, Drakeley C (2016). Application of serological tools and spatial analysis to investigate malaria transmission dynamics in highland areas of southwest Uganda. Am J Trop Med Hyg.

[13] Good MF, Kaslow DC, Miller LH (1998). Pathways and strategies for developing a malaria blood-stage vaccine. Annu Rev Immunol.

[14] Sepulveda N, Stresman G, White MT, Drakeley CJ (2015). Current mathematical models for analyzing anti-malarial antibody data with an eye to malaria elimination and eradication. J Immunol Res..

[15] Arnold BF, Priest JW, Hamlin KL, Moss DM, Colford JM, Lammie PJ (2014). Serological measures of malaria transmission in Haiti: comparison of longitudinal and cross-sectional methods. PLoS ONE.

[16] Egan AF, Morris J, Barnish G, Allen S, Greenwood BM, Kaslow DC (1996). Clinical immunity to *Plasmodium falciparum* malaria is associated with serum antibodies to the 19-kDa C-terminal fragment of the merozoite surface antigen, PfMSP-1. J Infect Dis.

[17] Akpogheneta OJ, Duah NO, Tetteh KKA, Dunyo S, Lanar DE, Pinder M (2008). Duration of naturally acquired antibody responses to blood-stage *Plasmodium falciparum* is age dependent and antigen specific. Infect Immun.

[18] Udhayakumar V, Kariuki S, Kolczack M, Girma M, Roberts JM, Oloo AJ (2001). Longitudinal study of natural immune responses to the *Plasmodium falciparum* apical membrane antigen (AMA-1) in a holoendemic region of malaria in western Kenya: asembo Bay Cohort Project VIII. Am J Trop Med Hyg.

[19] Bretscher MT, Supargiyono S, Wijayanti MA, Nugraheni D, Widyastuti AN, Lobo NF (2013). Measurement of *Plasmodium falciparum* transmission intensity using serological cohort data from Indonesian schoolchildren. Malar J..

[20] Greenhouse B, Ho B, Hubbard A, Njama-Meya D, Narum DL, Lanar DE (2011). Antibodies to *Plasmodium falciparum* antigens predict a higher risk of malaria but protection from symptoms once parasitemic. J Infect Dis.

[21] White MT, Griffin JT, Akpogheneta O, Conway DJ, Koram KA, Riley EM (2014). Dynamics of the antibody response to *Plasmodium falciparum* infection in African children. J Infect Dis.

[22] Bejon P, Williams TN, Liljander A, Noor AM, Wambua J, Marsh K (2010). Stable and unstable malaria hotspots in longitudinal cohort studies in Kenya. PLoS Med..

[23] Kramer RA, Mboera LEG, Senkoro K, Lesser A, Shayo EH, Paul CJ (2014). A randomized longitudinal factorial design to assess malaria vector control and disease management interventions in rural Tanzania. Int J Environ Res Public Health..

[24] Burghaus PA, Holder AA (1994). Expression of the 19-kilodalton carboxy-terminal fragment of the *Plasmodium falciparum* merozoite surface protein-1 in Escherichia coli as a correctly folded protein. Mol Biochem Parasitol.

[25] Hodder AN, Crewther PE, Anders RF (2001). Specificity of the protective antibody response to apical membrane antigen 1. Infect Immun.

[26] Laird NM, Ware JH (1982). Random-effects models for longitudinal data. Biometrics.

[27] Tibshirani R (2011). Regression shrinkage and selection via the lasso: a retrospective. J R Stat Soc Ser B..

[28] Muench H (1959). Catalytic models in epidemiology.

[29] Austin PC, Steyerberg EW (2012). Interpreting the concordance statistic of a logistic regression model: relation to the variance and odds ratio of a continuous explanatory variable. BMC Med Res Methodol.

[30] Bosomprah S (2014). A mathematical model of seropositivity to malaria antigen, allowing seropositivity to be prolonged by exposure. Malar J..

[31] Yman V, White MT, Rono J, Arcà B, Osier FH, Troye- M (2016). Antibody acquisition models: a new tool for serological surveillance of malaria transmission intensity. Sci Rep..

[32] Wipasa J, Suphavilai C, Okell LC, Cook J, Corran PH, Thaikla K (2010). Long-lived antibody and B cell memory responses to the human malaria parasites, *Plasmodium falciparum* and *Plasmodium vivax*. PLoS Pathog.

[33] Wong J, Hamel MJ, Drakeley CJ, Kariuki S, Shi YP, Lal AA (2014). Serological markers for monitoring historical changes in malaria transmission intensity in a highly endemic region of Western Kenya, 1994-2009. Malar J..

[34] Sepúlveda N, Paulino CD, Drakeley C (2015). Sample size and power calculations for detecting changes in malaria transmission using antibody seroconversion rate. Malar J..

[35] Struik SS, Riley EM (2004). Does malaria suffer from lack of memory?. Immunol Rev.

[36] Cavanagh DR, Elhassan IM, Roper C, Robinson VJ, Giha H, Holder AA (1998). A longitudinal study of type-specific antibody responses to *Plasmodium falciparum* merozoite surface protein-1 in an area of unstable malaria in Sudan. J Immunol..

[37] Kinyanjui SM, Bull P, Newbold CI, Marsh K (2003). Kinetics of antibody responses to *Plasmodium falciparum*-infected erythrocyte variant surface antigens. J Infect Dis.

[38] Kinyanjui SM, Conway DJ, Lanar DE, Marsh K (2007). IgG antibody responses to *Plasmodium falciparum* merozoite antigens in Kenyan children have a short half-life. Malar J..

[39] Raghunathan TE (2004). What do we do with missing data? Some options for analysis of incomplete data. Annu Rev Public Health.

[40] Pothin E, Ferguson NM, Drakeley CJ, Ghani AC (2016). Estimating malaria transmission intensity from *Plasmodium falciparum* serological data using antibody density models. Malar J..

[41] Bousema T, Okell L, Felger I, Drakeley C (2014). Asymptomatic malaria infections: public health relevance. Nat Rev Microbiol.

[42] Cook J, Speybroeck N, Sochanta T, Somony H, Sokny M, Claes F (2012). Sero-epidemiological evaluation of changes in *Plasmodium falciparum* and *Plasmodium vivax* transmission patterns over the rainy season in Cambodia. Malar J..

[43] van den Hoogen LL, Griffin JT, Cook J, Sepúlveda N, Corran P, Conway DJ (2015). Serology describes a profile of declining malaria transmission in Farafenni, The Gambia. Malar J..

[44] Baum E, Sattabongkot J, Sirichaisinthop J, Kiattibutr K, Jain A, Taghavian O (2016). Common asymptomatic and submicroscopic malaria infections in Western Thailand revealed in longitudinal molecular and serological studies: a challenge to malaria elimination. Malar J.

